# Enthalpies of Formation of (Cu,Ni)_3_Sn, (Cu,Ni)_6_Sn_5_-HT and (Ni,Cu)_3_Sn_2-_HT

**DOI:** 10.1007/s11669-014-0305-y

**Published:** 2014-04-12

**Authors:** C. Schmetterer, M. Rodriguez-Hortala, H. Flandorfer

**Affiliations:** 1 Forschungszentrum Jülich, IEK-2, 52425 Jülich, Germany; 2 Department of Inorganic Chemistry (Materials Chemistry), University of Vienna, Waehringerstr. 42, 1090 Vienna, Austria; 3 Institute of Materials Science and Technology, Vienna University of Technology, Karlsplatz 13, 1040 Vienna, Austria

**Keywords:** enthalpy of formation, experimental thermodynamics, intermetallics, metallic alloys, thermodynamic properties

## Abstract

Standard enthalpies of formation of ternary phases in the Cu-Ni-Sn system were determined along sections at 25, 41 and 45.5 at.% Sn applying tin solution drop calorimetry. Generally, the interaction of Ni with Sn is much stronger than that of Cu with Sn. Along all sections the enthalpy of formation changes almost linearly with the mutual substitution of Cu and Ni within the respective homogeneity ranges. Thus no additional ternary interaction promoting the formation of further Cu-Ni-Sn phases can be assumed. The results are discussed and compared with literature values relevant to this system.

## Introduction

Within the efforts of the electronics industry to develop a more environmentally friendly and less toxic technology, e.g. the switch to lead-free solders, the ternary Cu-Ni-Sn system assumed a key role, due to the focus on mostly Cu and Sn-bearing solders and Ni as a surface coating on the metallization. In recent years a number of investigations were carried out to determine the phase equilibria in this system that are the basis for targeted material design, especially for more sophisticated solder related technologies like transient liquid phase bonding. However, the elements involved are characterized by quite different melting temperatures and reliable experimental phase diagram investigations are hampered by uncertainties concerning the establishment of equilibrium at temperatures relevant for soldering in the high melting parts.

By combining experiments at higher temperatures with thermodynamic modelling of the phase equilibria (CALPHAD method) these difficulties can be overcome. This is a semi-empirical method and thus relies on available thermodynamic data. The phases usually formed in solder joints are Cu_6_Sn_5_, Ni_3_Sn_4_ and after long term annealing Cu_3_Sn. The enthalpies of formation of these phases (as well as a number of others) were determined by Flandorfer et al.[1]using drop-solution calorimetry and discussed together with other results from literature. Their work was limited to the binary compounds, but it is known from phase diagram studies that the high temperature (HT) phases of Cu_3_Sn and Ni_3_Sn (both DO_3_, BiF_3_ structure type) form a temperature dependent mutual solid solution in the ternary Cu-Ni-Sn system.[2] Such a complete mutual solution was not found for the Cu_6_Sn_5_ and Ni_3_Sn_2_ HT phases (both partially filled InNi_2_ type structures), but both of them dissolve considerable amounts of the respective third element.

In contrast to the binary compounds,[1] the enthalpies of formation of their ternary solid solutions have so far not been determined experimentally. First principles studies on Cu_6_Sn_5_ and Cu_6−*x*_Ni_*x*_Sn_5_ were conducted by Ghosh and Asta[3] and Yu et al.,[4] respectively. In the latter work four different substitutions of Cu by Ni in the Cu_6_Sn_5_-low temperature crystal structure were investigated. In all cases the enthalpy of formation values indicated a stabilization of the compound by the admixture of Ni. Cu_4_Ni_2_Sn_5_ was found to be the most stable one among these.

It was therefore considered of interest to provide experimental enthalpies of formation for several compositions within the homogeneity ranges of the various phases in the ternary Cu-Ni-Sn system using drop solution calorimetry.

## Materials and Methods

Ternary samples were prepared along three sections at 25 at.% Sn from Ni_3_Sn to Cu_3_Sn, at 41 at.% Sn starting from Ni_3_Sn_2_ and at 45.5 at.% Sn starting from Cu_6_Sn_5_. Their compositions can be found in Table 1. The continuous solid solution between Ni_3_Sn HT and Cu_3_Sn HT is strongly temperature dependent and does not appear as such at a single temperature (in isothermal sections of the phase diagram) due to the different thermal stability ranges of the binary phases. Therefore, at 973 K the phases Ni_3_Sn LT, τ1 or Cu_3_Sn HT were found in samples P2-P8, as shown in Table 1 (see also Ref 2). Ni_3_Sn_2_ HT and Cu_6_Sn_5_ HT even at varying temperature do not form a continuous solid solution. Furthermore, they differ in their Ni:Sn and Cu:Sn ratios according to the binary phase diagrams which resulted in the chosen placement of the samples.

**Table 1:** Sample composition, measurement details and enthalpy of formation results

Sample	Composition			Phase present after anneling	Crystal structure details			Annealing temperature, K	Experiment temperatures			$\Delta_{\text{Sol}} \bar{H}^{\infty }$, kJ/mol	$\Delta_{\text{f}} H^{298}$	
Number	Cu, at.%	Ni, at.%	Sn,at.%		Space group	Structure type	Lattice constants pm		Drop *T*, K	Measurement *T*, K			Value, kJ/mol atom	Error ±, kJ/mol atom
P2	60	15	25	Cu_3_Sn HT (Cu) traces	*Fm-3* *m * *Fm-3* *m*	BiF_3_ Cu	598.658(6) 359.3(1)	973	304		873	30.6	−11.7	1
P3	50	25	25	Cu_3_Sn HT	*Fm-3* *m*	BiF_3_	596.752(5)	973	310		873	27.9	−14.1	1
P4	40	35	25	Cu_3_Sn HT	*Fm-3* *m*	BiF _3_	595.194(8)	973	312		873	25	−16.2	1
P5	30	45	25	Cu_3_Sn HT	*Fm-3* *m*	BiF _3_	593.575(9)	973	304		873	22.1	−18.3	1
P7	12	63	25	τ1 τ1‘	*Cmcm* *Cmcm*	β-Cu_3_Ti β-Cu_3_Ti	537.6/429.6/451.0 582.8/415.7/429.5	973	307		873	16.4	−21.6	1
P8	5	70	25	Ni_3_Sn LT	*P6/3mmc*	Mg_3_Cd	529.98/426.092	973	308		873	14.6	−23.3	1
P9	30	29	41	Ni_3_Sn_2_ HT	*P6/3mmc*	InNi_2_	415.628/515.366	973	307		873	33.2	−22.1	1
P10	20	39	41	Ni_3_Sn_2_ HT	*P6/3mmc*	InNi_2_	413.455/518.125	973	304		873	32.3	−26.1	1
P11	10	49	41	Ni_3_Sn_2_ HT	*P6/3mmc*	InNi_2_	411.799/518.894	973	304		873	30.1	−28.9	1
P12	27.5	27	45.5	Cu_6_Sn_5_ HT (Sn) traces	*P6/3mmc* *I4/amd*	InNi_2_ β-Sn	415.060(3)/510.604(5) 583.222(9)/318.188(7)	673	304		873	30.2	−18.2	2
P13	37.5	17	45.5	Cu_6_Sn_5_ HT (Sn) traces	*P6/3mmc* *I4/amd*	InNi_2_ β-Sn	420.604(3)/510.827(5) 583.23(2)/318.13(2)	673	304		873	33.3	−16.2	2

The samples were prepared from Cu (99.98%, Goodfellow, Cambridge, UK), Ni (99.98%) and Sn (99.9985%, both Alfa Johnson Matthey, Karlsruhe, Germany). Before use, Cu was treated under H_2_ flow at 473 K in order to remove oxide layers, while the other metals were used as received.

The weighed pure metals were fused together in an arc furnace in Ar atmosphere. The sample pellets were re-melted three to four times and turned upside down in order to ensure homogeneity. After alloying, the samples were sealed in evacuated quartz glass tubes and annealed at 973 and 673 K, respectively—see Table 1. The samples were then quenched in cold water.

Prior to the calorimetric measurements, the samples were characterised by means of x-ray diffraction (XRD) which was carried out on a Bruker D8 diffractometer in Bragg-Brentano geometry (Bruker AXS, Karlsruhe, Germany) using Cu Kα_1_ radiation and an exposure time of two hours. The obtained diffractograms were analyzed using the Topas 3 software (Bruker AXS, Karlsruhe, Germany) in order to check that the desired phase had formed and to determine possible traces of impurity phases.

Drop solution calorimetry used to determine the enthalpies of formation was done on a Calvet-type micro calorimeter (HT1000 Setaram, Caluire, France). This calorimeter consists of a twin cell for sample and reference. Each cell is surrounded by a thermopile of more than 200 thermocouples. It is heated by a wire wound resistance furnace suitable for operating temperatures up to 1273 K. A self-made drop device was used for dropping up to 30 sample pieces during each experiment, while measurement control and data recording were done using the software LabView and HiQ (both National Instruments). A more detailed description of the setup was given by Flandorfer et al.[5]

The experimental setup consisted of a quartz glass tube inserted into the measurement cell, which housed a BN (boronitride) crucible containing the Sn-bath (4-12 g). Before each measurement, the whole apparatus was flushed several times with high purity Ar (99.999% with Oxisorb cleaning system) and a constant Ar flow of 30 mL/min was established during the measurement. Small pieces (10-40 mg) of the pure transition metals or the alloys were dropped from the automatic drop device at the drop temperature (DT; see Table 1) into the Sn-bath kept at the measurement temperature (MT = 873 K). Calibration was done at the end of each measurement by dropping pieces of α-Al_2_O_3_ standard (~30 mg each) supplied by National Institute of Standards and Technology (NIST, Gaithersburg, MD, USA). Temperature measurement was done using thermocouples and thermo-resistors with accuracy higher than ±1 K. For the evaluation the average values of DT and MT of each drop in an experiment were taken.

The quantity measured in the experiments is the enthalpy of solution, $\Delta_{\text{Sol}} \bar{H} (A )$, where *A* represents either a pure metal or a compound. This was obtained by successive additions of the respective material to the bath of pure Sn or Sn-alloy (after the first drop). Recalculation of the measured heat effects to a basis of one mole of dropped substance yielded the approximate partial heat of solution $\Delta_{\text{Sol}} \bar{H} (A )$ for dropping a sample piece from DT to the tin bath at MT. This is valid only if the masses of dropped substance are very small compared to the mass of material in the crucible.

**Fig.1:**
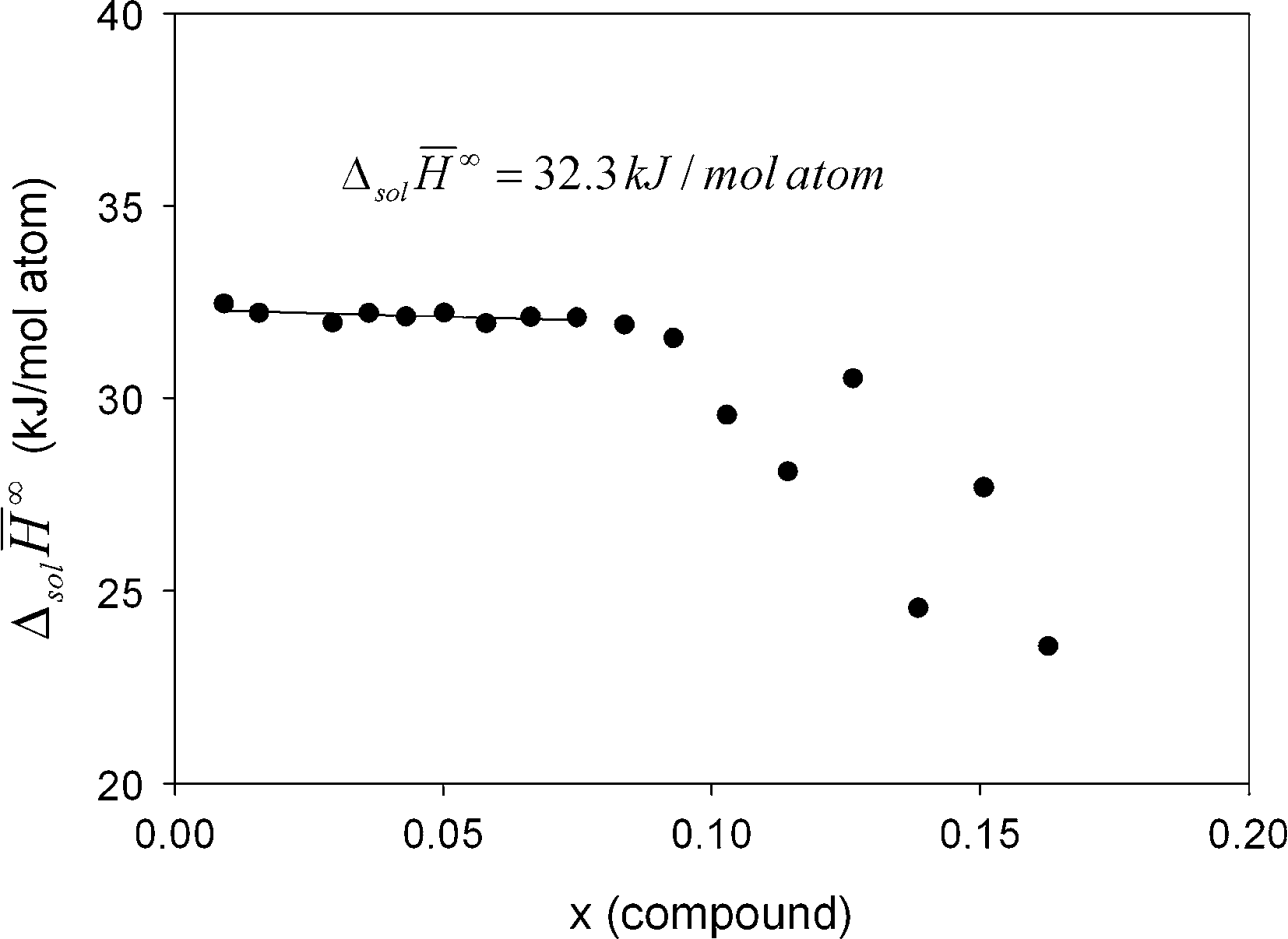
Molar enthalpies of solution of alloy P10, Cu_20_Ni_39_Sn_41_, in a liquid tin bath at 873 K. The extrapolated value at infinite dilution is given. The strong decrease in the enthalpy of solution above *x* = 0.08 is due to the formation of an intermetallic compound (probably Ni_3_Sn_4_). This range of the data was therefore not used to determine the limiting enthalpy of solution

**Fig.2:**
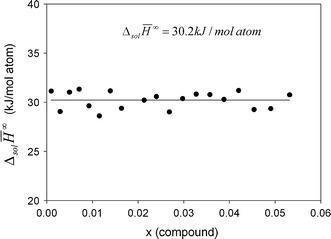
Molar enthalpies of solution of alloy P12, Cu_27.5_Ni_27_Sn_45.5_, in a liquid tin bath at 873 K. The extrapolated value at infinite dilution is given

In order to derive the enthalpy of formation of a compound, $\Delta_{\text{f}} H^{298}$, the limiting partial enthalpies of solution at infinite dilution $\Delta_{\text{Sol}} \bar{H}^{\infty } (A )$ need to be known. They are obtained by extrapolating the values of $\Delta_{\text{Sol}} \bar{H} (A )$ of each drop made during an experiment to a mole fraction of zero. In the composition range very close to pure tin $\Delta_{\text{Sol}} \bar{H} ( {\text{A)}}$ can be treated as a linear function of composition. If this condition is not fulfilled over the entire composition range, only the linear part can be used or another function needs to be used for extrapolation. Examples for both cases are shown in Fig. 1 and 2 for samples P10 and P12, respectively.

The enthalpy of formation was then derived as the difference between the limiting enthalpies of solution at infinite dilution of the pure elements and the respective alloy in liquid Sn as expressed by Eq 1:

1 $$\Delta_{\text{f}} H^{298} = x \cdot \Delta_{\text{Sol}} \bar{H}^{\infty } ( {\text{Cu)}} + y \cdot \Delta_{\text{Sol}} \bar{H}^{\infty } (Ni )+ z \cdot \Delta_{\text{Sol}} \bar{H}^{\infty } ( {\text{Sn)}} - \Delta_{\text{Sol}} \bar{H}^{\infty } ( {\text{Cu}}_{x} {\text{Ni}}_{y} {\text{Sn}}_{z} ) ,$$

- $\Delta_{\text{f}} H^{298}$ is the enthalpy of formation of the intermetallic compound at 298 K with the pure solid metals being the reference state.
- *x*, *y* and *z* are the mole fractions of Cu, Ni and Sn
- $\Delta_{\text{Sol}} \bar{H}^{\infty } (A )$ are the limiting enthalpies of solution at infinite dilution of the pure elements or the compound in liquid Sn.

The $\Delta_{\text{Sol}} \bar{H}^{\infty }$ values contain the following contributions:

2 $$\begin{gathered} \Delta_{\text{Sol}} \bar{H}^{\infty } (A )= \int\limits_{\text{DT}}^{{{\text{Tm}}(A)}} {{\text{Cp(}}A,s )\,dT + } \int\limits_{{{\text{Tm(}}A )}}^{\text{MT}} {{\text{Cp(}}A,l )\,dT + } \,\Delta_{\text{fus}} H (A,{\text{Tm)}} + \Delta_{\text{mix}} \bar{H}^{\infty } (A )\,{\text{for}}\,MT > {\text{Tm}} \hfill \\ \Delta_{\text{Sol}} \bar{H}^{\infty } (A )= \int\limits_{\text{DT}}^{\text{MT}} {{\text{Cp(}}A,s )\,dT + } \,\Delta_{\text{fus}} H (A,MT )+ \Delta_{\text{mix}} \bar{H}^{\infty } (A ) {\text{ for}}\,MT\, < \,{\text{Tm}} \hfill \\ \end{gathered}$$

- Integral over Cp—relative enthalpies of heating of solid and liquid component over the respective temperature ranges
- Enthalpy of fusion—$\Delta_{\text{fus}} H$
- Limiting partial enthalpy of mixing of the component or alloy with liquid Sn—$\Delta_{\text{mix}} \bar{H}^{\infty }$.

The terms in Eq 2 can either be taken from available literature or need to be determined in the course of the experiments. $\Delta_{\text{mix}} \bar{H}^{\infty }$ is zero for dropping Sn into a Sn-bath so that $\Delta_{\text{Sol}} \bar{H}^{\infty } ( {\text{Sn)}}$ can simply be calculated from available SGTE data[6] and does not need to be determined experimentally. Equation 1 is valid only under the assumption that the limiting enthalpies of mechanical mixtures of Cu and Ni in liquid Sn are a linear combination of the limiting enthalpies of the pure compounds. The error of the measured $\Delta_{\text{Sol}} \bar{H}^{\infty }$ and of the calculated final Δ_f_ *H* was determined from the experimental scatter and is smaller than ±1 kJ/mol unless otherwise stated.

## Results and Discussion

**Fig. 3:**
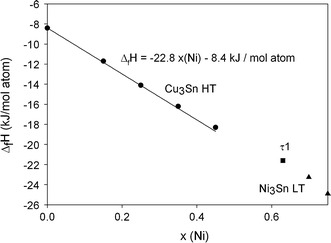
Enthalpies of formation of alloys at a constant Sn-content of 25 at.% (samples P2-P8). Circles represent the Cu_3_Sn HT phase, squares the τ1 ternary compound and triangles the Ni_3_Sn LT phase

**Fig. 4:**
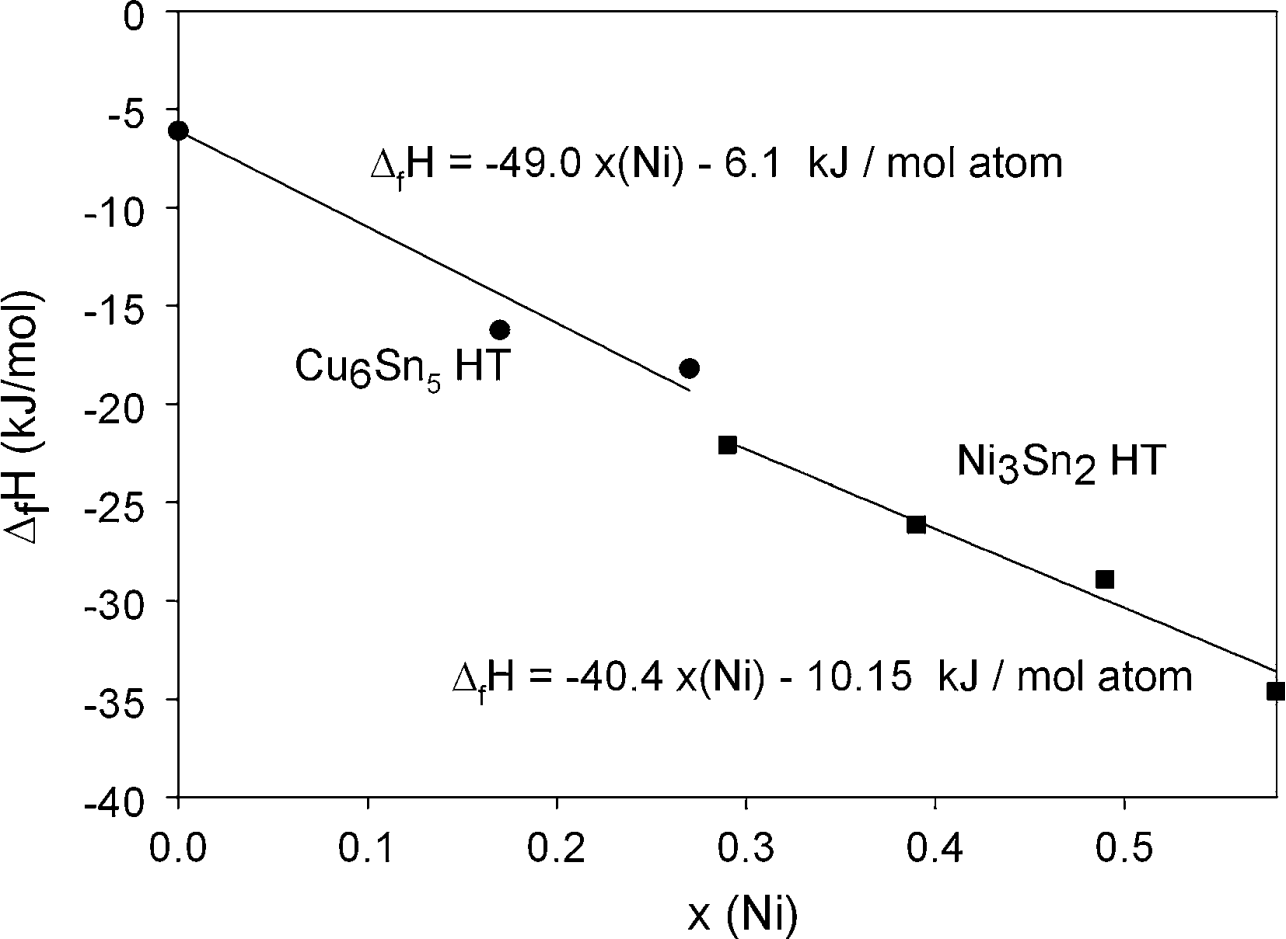
Enthalpies of formation of alloys at a constant Sn-contents of 41 at.% (squares: Ni_3_Sn_2_ HT, samples P9-P11) and 45.5 at.% (circles: Cu_6_Sn_5_ HT, samples P12-P13)

Values of $\Delta_{\text{Sol}} \bar{H}^{\infty }$ and $\Delta_{\text{f}} H^{298}$ for the investigated alloys are presented in Table 1. A graphical representation of the $\Delta_{\text{f}} H^{298}$ values is given in Fig. 3 (25 at.% Sn section) and Fig. 4 (41 and 45.5 at.% Sn sections). The values for the limiting binary compounds were taken from Flandorfer et al.[1] Within limits of error in all sections, a linear dependency between the enthalpy of formation and the change in composition (change in the Cu/Ni ratio) is observed. Interestingly, at 25 at.% Sn, this linear trend is unaffected by the presence of different phases: τ1 and Ni_3_Sn LT at 53 and 70 at.% Ni, respectively, and Cu_3_Sn HT in the remaining samples throughout this section. The occurrence of these phases is in agreement with the phase diagram.[2] The data-fit shown in Fig. 3 has been done only for the Cu_3_Sn HT values, while no data-fit was made for the other two phases due to the limited availability of ternary data points for these phases.

It has to be noted that sample P2, Cu_60_Ni_15_Sn_25_, contained trace amounts of (Cu), which can be considered negligible (only 2% according to XRD), while sample P7, Cu_12_Ni_63_Sn_25_, contains the stable and metastable variants of τ1 and τ1′ in equal amounts. Both phases crystallize in the β-Cu_3_Ti type structure and only differ in the free parameters of the atom positions and variations of the lattice parameters.[2] Any influence on the enthalpy of formation should therefore be negligible.

Another sample, Cu_68_Ni_7_Sn_25_, had initially been prepared, but in none of the preparation runs the Cu_3_Sn HT phase could be fully retained. Instead, this sample partially decomposed into Cu_41_Sn_11_ (40%) and (Cu) (20%); its values are therefore not given in Table 1. However, the enthalpy of formation of this sample was determined to be −9.8 kJ/mol atom, which corresponds to the observed linear trend.

From these observations, it can be concluded that the enthalpy of formation of Cu-Ni-Sn phases at 25 at.% Sn predominantly depends on the replacement of Cu by Ni rather than on the actual type of phase present in the sample. Indeed, the crystal structures of the phases found along this section show some clear relationships. The most obvious relation can be found between the cubic DO_3_ type (Cu,Ni)_3_Sn HT phase and the orthorhombic τ1 phase which are both composed of (Cu,Ni)- and Sn-chains. While the arrangements of these two kinds of chains is regular in the Cu_3_Sn HT phase according to the cubic symmetry, an offset between the chains along the c-axis can be noticed in the τ1 phase which breaks the cubic symmetry and necessitates the orthorhombic setting. Nevertheless, the coordination of the individual atoms remains similar in the two phases which indicates similar (Cu,Ni)-Sn bonds. As the local environment of the atoms appears to be similar in all phases, the exchange of Cu and Ni has the main influence on the enthalpy of formation rather than the actual phase formed.

An equally linear trend was found for the Ni_3_Sn_2_ HT and Cu_6_Sn_5_ HT phases (samples P9-P11, and P12, P13), respectively. As Ni_3_Sn_2_ HT is a congruently melting compound, single phase samples could easily be prepared. On the contrary, despite having the same crystal structure, Cu_6_Sn_5_ HT is formed in a peritectic reaction in the binary Cu-Sn system. In the ternary system, too, it was therefore not possible to obtain single phase samples despite prolonged annealing. Samples P12 and P13 contain trace amounts of (Sn) according to XRD which are considered negligible. Nevertheless, a larger error of ±2 kJ/mol atom is given in the results for these samples.

Yu et al.[4] calculated enthalpy of formation values for the admixture of Ni to the Cu_6_Sn_5_ LT phase (see Table 2) using first principles calculations. Their values are in general lower than those reported in the present study and well established values in the literature,[1] but are valid for 0 K and the low temperature phase. However, they show the same trend as the data in the present work valid at 298 K for the HT phase, i.e. increasing exothermic enthalpy of formation by the admixture of Ni to Cu_6_Sn_5_ HT.

**Table 2:** Enthalpy of formation values of (Cu,Ni)_6_Sn_5_ LT as reported in Ref 4

	Composition			Δ_f_ *H* at 0 K, kJ/mol atom	Cu atom site substituted by Ni as in Ref [4]
	Cu, at.%	Ni, at.%	Sn, at.%		
Cu_6_Sn_5_	54.55	0.00	45.45	−26.32	
Cu_4_Ni_2_Sn_5_	36.36	18.18	45.45	−53.73	8f (Cu1)
Cu_4_Ni_2_Sn_5_	36.36	18.18	45.45	−59.12	8f (Cu2)
Cu_5_NiSn_5_	45.45	9.09	45.45	−50.03	4a (Cu3)
Cu_5_NiSn_5_	45.45	9.09	45.45	−51.91	4e (Cu4)

The enthalpy of formation can be regarded as a measure for the stability of a compound, since the entropy of formation is usually very small and influences the stability only at very HT.[7] According to the present results, the addition of Ni enhances the stability of the Cu-Sn compounds whereas the addition of Cu to Ni-Sn systems has the reverse effect. A stronger electronic interaction of Ni with Sn due to the even number of valence electrons of both elements is assumed to be responsible for this behaviour. A similar effect can also be observed in the enthalpies of mixing of liquid state in the systems Cu-Sn and Ni-Sn, where Flandorfer et al.[8] reported minima of −3.6 kJ/mol at *x*_Cu_ = 0.73 and −20.6 kJ/mol at *x*_Ni_ = 0.60, respectively at 1373 K. This assumption is supported by the occurrence of short range order in the liquid state.
